# [μ-*N* ^1^,*N* ^2^-Bis(pyridin-2-yl)hydrazine-1,2-dicarbothio­amidato]bis­[chlorido­copper(II)]

**DOI:** 10.1107/S1600536812048659

**Published:** 2012-12-12

**Authors:** Yang Liu, Bingguang Zhang, Kejian Deng

**Affiliations:** a College of Chemistry and Material Science, South-Central University for Nationalities, Wuhan, Hubei 430074, People’s Republic of China

## Abstract

The binuclear title compound, [Cu_2_(C_12_H_10_N_6_S_2_)Cl_2_], possesses twofold rotational symmetry. The Cu^II^ atom occupies a four-coordinate pseudo-tetra­hedral environment bound to one S atom, one imine N atom and one pyridine N atom from the *N* ^1^,*N* ^2^-bis­(pyridin-2-yl)hydrazine-1,2-dicarbo­thio­amidate ligand, and one Cl^−^ anion. The metal atoms are connected *via* the bis-tridentate ligand into a binuclear structure. The mol­ecule is bow-shaped with the pyridine rings inclined to one another by 51.56 (14)°. In the crystal, N—H⋯Cl hydrogen bonds lead to the formation of ribbons propagating along [001]. These ribbons are connected *via* C—H⋯Cl, C—H⋯S and π–π inter­actions [centroid–centroid distance = 3.6146 (19) Å], leading to the formation of a three-dimensional structure.

## Related literature

For the biological activity of thio­semicarbazides and their metal complexes, see: West *et al.* (1993 ▶). For related structures, see: Wang *et al.* (2011 ▶); Yamin & Yusof (2003 ▶); Akinchan *et al.* (2002 ▶). For the synthesis of the ligand, see: Szecsenyi *et al.* (2006 ▶).
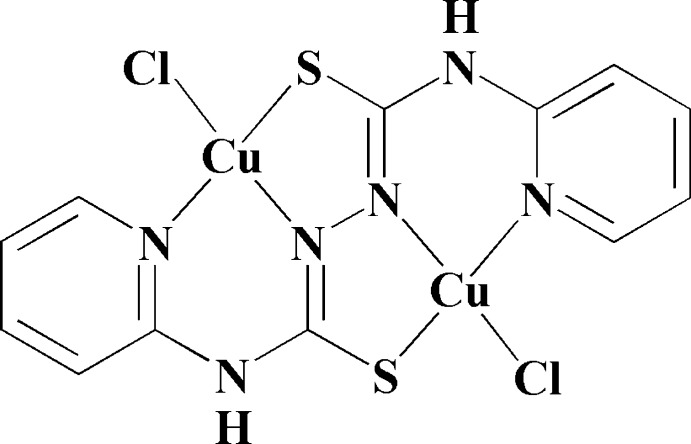


## Experimental

### 

#### Crystal data

[Cu_2_(C_12_H_10_N_6_S_2_)Cl_2_]
*M* *_r_* = 500.36
Monoclinic, 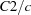

*a* = 15.825 (3) Å
*b* = 7.6190 (13) Å
*c* = 15.082 (4) Å
β = 118.179 (2)°
*V* = 1602.9 (6) Å^3^
*Z* = 4
Mo *K*α radiation
μ = 3.26 mm^−1^
*T* = 293 K
0.32 × 0.28 × 0.27 mm

#### Data collection

Bruker SMART APEX CCD diffractometer
Absorption correction: multi-scan (*SADABS*; Bruker, 2003 ▶) *T*_min_ = 0.422, *T*_max_ = 0.474
4270 measured reflections
1561 independent reflections
1445 reflections with *I* > 2σ(*I*)
*R*_int_ = 0.018

#### Refinement

*R*[*F* ^2^ > 2σ(*F* ^2^)] = 0.024
*wR*(*F* ^2^) = 0.068
*S* = 1.07
1561 reflections
110 parameters
H-atom parameters constrained
Δρ_max_ = 0.73 e Å^−3^
Δρ_min_ = −0.34 e Å^−3^

### 

Data collection: *SMART* (Bruker, 2003 ▶); cell refinement: *SAINT-Plus* (Bruker, 2003 ▶); data reduction: *SAINT-Plus*; program(s) used to solve structure: *SHELXS97* (Sheldrick, 2008 ▶); program(s) used to refine structure: *SHELXL97* (Sheldrick, 2008 ▶); molecular graphics: *SHELXTL* (Sheldrick, 2008 ▶); software used to prepare material for publication: *SHELXTL*.

## Supplementary Material

Crystal structure: contains datablock(s) global, I. DOI: 10.1107/S1600536812048659/su2393sup1.cif

Structure factors: contains datablock(s) I. DOI: 10.1107/S1600536812048659/su2393Isup2.hkl

Additional supplementary materials:  crystallographic information; 3D view; checkCIF report

## Figures and Tables

**Table 1:** Selected bond lengths (Å)

Cu1—Cl1	2.2619 (10)
Cu1—S2	2.2295 (9)
Cu1—N1	1.986 (2)
Cu1—N3^i^	1.961 (3)

Symmetry code: (i) 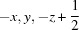.

**Table 2:** Hydrogen-bond geometry (Å, °)

*D*—H⋯*A*	*D*—H	H⋯*A*	*D*⋯*A*	*D*—H⋯*A*
N2—H2*A*⋯Cl1^ii^	0.86	2.70	3.507 (2)	156
C2—H2⋯Cl1^iii^	0.93	2.77	3.482 (3)	134
C5—H5⋯S2^iv^	0.93	2.82	3.425 (3)	124

Symmetry codes: (ii) 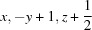; (iii) 
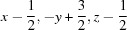
; (iv) 
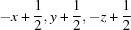
.

**Fig. 1.**
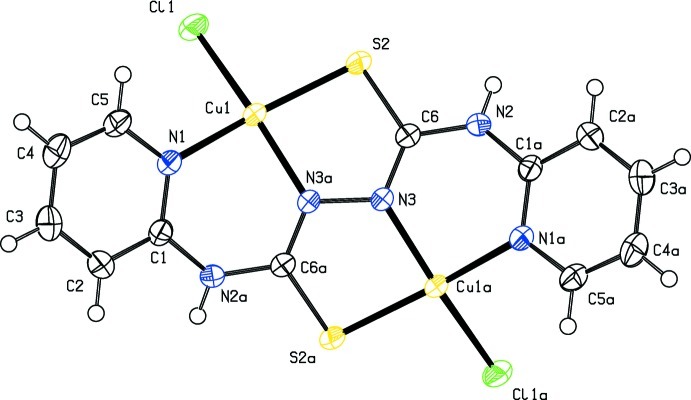
The molecular structure of title compound, with the atom numbering. The displacement ellipsoids are drawn at the 50% probability level [symmetry code: (a) - x, y, - z + 1/2].

## supplementary crystallographic information

### Comment

Thiosemicarbazide and their metal complexes have attracked considerable interest due to their biological activities, such as antiviral, antibacterial, antimalarial, antifungal, and antitumoral activities (West *et al.*, 1993). Thiosemicarbazide are versatile ligands that can coordinate as neutral ligands or in the deprotonated form. They can also be used as flexible spacers with potential multiple binding sites to construct coordination polymers with multiple dimensions and various topologies. In the present paper, the synthesis and crystal structure of the title thiosemicarbazide binuclear copper(II) compound is reported.

The title compound possesses twofold rotational symmetry (Fig.1). Each Cu^II^ center occupies a four-coordinated pseudotetrahedral environment bound to one sulfur atom, one imine nitrogen atom, and one pyridine nitrogen atom from one *N*,*N'*-di(pyridin-2-yl)hydrazine-1,2-bis(carbothioamide) ligand, and one chlorine anion. The metal centres are connected *via* the hexadentate ligand into a binuclear structure. The molecule is bow-shaped. The thiosemicarbazide moiety (S2/N2(N3/N6) is twisted by 20.14 (13)° from the pyridine ring to which it is attached. The two thiosemicarbazide moieties, (S2/N2/N3/N6) and (S2A/N2A/N3A/N6A), are inclined to one another by 23.36 (13) °, while the pyrdine rings make a dihedral angle of 51.56 (14)°.

The Cu—S distance is 2.2295 (9) Å, and the Cu—N distances vary between 1.961 (3)–1.986 (2) Å. The C—S bond distances of 1.711 (3) Å are within the normal range for a C—S single bond, indicating that the thiosemicarbazide moieties adopt the thiol tautomeric form, acting as a doubly charged negative ligand. The C6—N distances of 1.311 (3)–1.366 (3) Å and the N3—N3A distance of 1.399 (3) Å are intermediate between formal single and double bonds, pointing to extensive electron delocalization over the entire ligand skeleton. This agrees well with the same distances observed in related compounds (Wang *et al.*, 2011; Yamin & Yusof, 2003; Akinchan *et al.*, 2002).

In the crystal, there are N-H···Cl hydrogen bonds, leading to the formation of ribbons propagating along [001], and C-H···Cl and C-H···S interactions (Table 1). The latter link the ribbons and together with π–π interactions lead to the formation of a three-dimensional structure [Cg1···Cg1^i^ 3.6146 (19) Å; perpendicular separation 3.5312 (11) Å; slippage 0.772 Å; Cg1 is the centroid of pyrdine ring N1/C1-C5; symmetry code: (i) -x, -y+2, -z].

### Experimental

The ligand (L), *N*,*N'*-di(pyridin-2-yl)hydrazine-1,2-bis(carbothioamide,) was prepared by the literature method (Szecsenyi *et al.*, 2006). L (0.05 mmol) was solved in DMF (5 ml) in a test tube, then an 8 ml solvent mixture of CH_3_OH and DMF (v/v = 1:1) was added as a buffer layer. A solution of CuCl_2_(0.10 mmol) in CH_3_OH (3 ml) was then carefully layered on top. The system was sealed and kept for a week, after which black block-like single crystals, suitable for X-ray analysis, were obtained. Anal. Calcd for C_12_H_10_Cl_2_Cu_2_N_6_S_2_: C 28.80, H 2.01, N16.80. Found: C 29.23; H, 2.40; N, 16.44.

### Refinement

The NH and C-bound H atoms were included in calculated positions and treated as riding atoms: N–H = 0.86 Å and C–H = 0.93 Å , with *U*_iso_(H) = 1.2*U*_eq_(N,C).

### Crystal data

**Table:**

[Cu_2_(C_12_H_10_N_6_S_2_)Cl_2_]	*F*(000) = 992
*M**_r_* = 500.36	*D*_x_ = 2.073 Mg m^−^^3^
Monoclinic, *C*2/*c*	Mo *K*α radiation, λ = 0.71073 Å
Hall symbol: -C 2yc	Cell parameters from 253 reflections
*a* = 15.825 (3) Å	θ = 2.9–29.5°
*b* = 7.6190 (13) Å	µ = 3.26 mm^−^^1^
*c* = 15.082 (4) Å	*T* = 293 K
β = 118.179 (2)°	Block, black
*V* = 1602.9 (6) Å^3^	0.32 × 0.28 × 0.27 mm
*Z* = 4	

### Data collection

**Table:**

Bruker SMART APEX CCD diffractometer	1561 independent reflections
Radiation source: fine-focus sealed tube	1445 reflections with *I* > 2σ(*I*)
Graphite monochromator	*R*_int_ = 0.018
φ and ω scans	θ_max_ = 26.0°, θ_min_ = 2.9°
Absorption correction: multi-scan (*SADABS*; Bruker, 2003)	*h* = −19→18
*T*_min_ = 0.422, *T*_max_ = 0.474	*k* = −9→7
4270 measured reflections	*l* = −18→17

### Refinement

**Table:**

Refinement on *F*^2^	Secondary atom site location: difference Fourier map
Least-squares matrix: full	Hydrogen site location: inferred from neighbouring sites
*R*[*F*^2^ > 2σ(*F*^2^)] = 0.024	H-atom parameters constrained
*wR*(*F*^2^) = 0.068	*w* = 1/[σ^2^(*F*_o_^2^) + (0.0374*P*)^2^ + 2.3083*P*] where *P* = (*F*_o_^2^ + 2*F*_c_^2^)/3
*S* = 1.07	(Δ/σ)_max_ < 0.001
1561 reflections	Δρ_max_ = 0.73 e Å^−^^3^
110 parameters	Δρ_min_ = −0.34 e Å^−^^3^
0 restraints	Extinction correction: *SHELXL97* (Sheldrick, 2008), Fc^*^=kFc[1+0.001xFc^2^λ^3^/sin(2θ)]^-1/4^
Primary atom site location: structure-invariant direct methods	Extinction coefficient: 0.0018 (3)

### Special details

**Table:**

Geometry. Bond distances, angles etc. have been calculated using the rounded fractional coordinates. All su's are estimated from the variances of the (full) variance-covariance matrix. The cell esds are taken into account in the estimation of distances, angles and torsion angles
Refinement. Refinement of *F*^2^ against ALL reflections. The weighted *R*-factor *wR* and goodness of fit *S* are based on *F*^2^, conventional *R*-factors *R* are based on *F*, with *F* set to zero for negative *F*^2^. The threshold expression of *F*^2^ > σ(*F*^2^) is used only for calculating *R*-factors(gt) *etc*. and is not relevant to the choice of reflections for refinement. *R*-factors based on *F*^2^ are statistically about twice as large as those based on *F*, and *R*- factors based on ALL data will be even larger.

### Fractional atomic coordinates and isotropic or equivalent isotropic displacement parameters (Å^2^)

**Table:**

	*x*	*y*	*z*	*U*_iso_*/*U*_eq_	
Cu1	0.12780 (2)	0.63445 (4)	0.20890 (2)	0.0266 (1)	
Cl1	0.25399 (4)	0.50248 (9)	0.20491 (5)	0.0355 (2)	
S2	0.18501 (4)	0.59737 (10)	0.37400 (5)	0.0336 (2)	
N1	0.07304 (14)	0.7259 (3)	0.06931 (14)	0.0267 (6)	
N2	0.08612 (15)	0.6577 (3)	0.46876 (15)	0.0297 (6)	
N3	−0.00303 (14)	0.6631 (3)	0.29480 (15)	0.0258 (6)	
C1	−0.02056 (17)	0.7259 (3)	0.00390 (18)	0.0265 (7)	
C2	−0.05668 (19)	0.7912 (4)	−0.09283 (19)	0.0356 (8)	
C3	0.0058 (2)	0.8611 (4)	−0.1223 (2)	0.0452 (10)	
C4	0.1031 (2)	0.8622 (4)	−0.0559 (2)	0.0434 (10)	
C5	0.13323 (19)	0.7951 (4)	0.03822 (19)	0.0340 (8)	
C6	0.07986 (17)	0.6433 (3)	0.37555 (18)	0.0248 (7)	
H2	−0.12210	0.78770	−0.13690	0.0430*	
H2A	0.13840	0.61870	0.51760	0.0360*	
H3	−0.01690	0.90740	−0.18660	0.0540*	
H4	0.14670	0.90750	−0.07490	0.0520*	
H5	0.19850	0.79710	0.08320	0.0410*	

### Atomic displacement parameters (Å^2^)

**Table:**

	*U*^11^	*U*^22^	*U*^33^	*U*^12^	*U*^13^	*U*^23^
Cu1	0.0178 (2)	0.0406 (2)	0.0238 (2)	0.0017 (1)	0.0118 (1)	0.0002 (1)
Cl1	0.0250 (3)	0.0472 (4)	0.0381 (3)	0.0039 (3)	0.0181 (3)	−0.0055 (3)
S2	0.0188 (3)	0.0567 (4)	0.0270 (3)	0.0091 (3)	0.0123 (3)	0.0067 (3)
N1	0.0221 (10)	0.0354 (12)	0.0249 (10)	−0.0031 (8)	0.0131 (8)	−0.0023 (9)
N2	0.0190 (10)	0.0477 (13)	0.0217 (10)	0.0072 (9)	0.0090 (9)	0.0026 (9)
N3	0.0191 (10)	0.0392 (12)	0.0213 (9)	−0.0001 (8)	0.0113 (8)	0.0005 (8)
C1	0.0251 (12)	0.0323 (13)	0.0260 (11)	−0.0019 (10)	0.0152 (10)	−0.0031 (10)
C2	0.0294 (13)	0.0508 (17)	0.0245 (12)	−0.0022 (12)	0.0109 (11)	0.0020 (11)
C3	0.0462 (18)	0.064 (2)	0.0302 (14)	−0.0015 (14)	0.0221 (14)	0.0078 (13)
C4	0.0428 (17)	0.0564 (19)	0.0422 (16)	−0.0074 (13)	0.0294 (15)	0.0031 (13)
C5	0.0268 (13)	0.0445 (15)	0.0354 (13)	−0.0062 (11)	0.0186 (12)	−0.0029 (12)
C6	0.0204 (12)	0.0304 (13)	0.0252 (11)	0.0001 (9)	0.0121 (10)	0.0007 (9)

### Geometric parameters (Å, º)

**Table:**

Cu1—Cl1	2.2619 (10)	N3—N3^i^	1.399 (3)
Cu1—S2	2.2295 (9)	N2—H2A	0.8600
Cu1—N1	1.986 (2)	C1—C2	1.384 (4)
Cu1—N3^i^	1.961 (3)	C2—C3	1.369 (5)
S2—C6	1.711 (3)	C3—C4	1.385 (4)
N1—C1	1.337 (4)	C4—C5	1.366 (4)
N1—C5	1.352 (4)	C2—H2	0.9300
N2—C6	1.366 (3)	C3—H3	0.9300
N2—C1^i^	1.386 (4)	C4—H4	0.9300
N3—C6	1.311 (3)	C5—H5	0.9300
			
Cl1—Cu1—S2	94.32 (3)	N1—C1—C2	122.7 (3)
Cl1—Cu1—N1	94.39 (7)	N1—C1—N2^i^	120.3 (2)
Cl1—Cu1—N3^i^	159.80 (7)	C1—C2—C3	118.7 (3)
S2—Cu1—N1	166.41 (7)	C2—C3—C4	119.6 (3)
S2—Cu1—N3^i^	85.31 (6)	C3—C4—C5	118.2 (3)
N1—Cu1—N3^i^	90.08 (9)	N1—C5—C4	123.3 (3)
Cu1—S2—C6	96.01 (9)	N2—C6—N3	120.1 (3)
Cu1—N1—C1	123.98 (19)	S2—C6—N2	115.6 (2)
Cu1—N1—C5	118.58 (18)	S2—C6—N3	124.3 (2)
C1—N1—C5	117.4 (2)	C1—C2—H2	121.00
C1^i^—N2—C6	129.5 (2)	C3—C2—H2	121.00
Cu1^i^—N3—C6	124.48 (19)	C2—C3—H3	120.00
N3^i^—N3—C6	113.7 (2)	C4—C3—H3	120.00
Cu1^i^—N3—N3^i^	119.99 (16)	C3—C4—H4	121.00
C6—N2—H2A	115.00	C5—C4—H4	121.00
C1^i^—N2—H2A	115.00	N1—C5—H5	118.00
N2^i^—C1—C2	117.0 (3)	C4—C5—H5	118.00
			
Cl1—Cu1—S2—C6	−165.43 (8)	C5—N1—C1—N2^i^	−179.7 (2)
N3^i^—Cu1—S2—C6	−5.68 (11)	Cu1—N1—C5—C4	−179.7 (2)
Cl1—Cu1—N1—C1	138.8 (2)	C1^i^—N2—C6—S2	166.0 (2)
Cl1—Cu1—N1—C5	−42.3 (2)	C6^i^—N2^i^—C1—N1	26.4 (4)
N3^i^—Cu1—N1—C1	−21.5 (2)	C6^i^—N2^i^—C1—C2	−154.0 (3)
N3^i^—Cu1—N1—C5	157.5 (2)	C1^i^—N2—C6—N3	−14.5 (4)
Cl1—Cu1—N3^i^—N3	94.0 (2)	N3^i^—N3—C6—N2	173.7 (2)
Cl1—Cu1—N3^i^—C6^i^	−69.8 (3)	Cu1^i^—N3—C6—S2	157.76 (14)
S2—Cu1—N3^i^—N3	4.18 (18)	Cu1^i^—N3—C6—N2	−21.7 (3)
S2—Cu1—N3^i^—C6^i^	−159.6 (2)	C6—N3—N3^i^—Cu1	0.2 (3)
N1—Cu1—N3^i^—N3	−163.03 (19)	C6—N3—N3^i^—C6^i^	165.6 (2)
N1—Cu1—N3^i^—C6^i^	33.2 (2)	Cu1^i^—N3—N3^i^—Cu1	−165.23 (11)
Cu1—S2—C6—N2	−171.77 (17)	N3^i^—N3—C6—S2	−6.9 (3)
Cu1—S2—C6—N3	8.8 (2)	N1—C1—C2—C3	−1.0 (4)
C1—N1—C5—C4	−0.7 (4)	N2^i^—C1—C2—C3	179.4 (3)
Cu1—N1—C1—C2	179.7 (2)	C1—C2—C3—C4	1.1 (4)
C5—N1—C1—C2	0.8 (4)	C2—C3—C4—C5	−1.0 (5)
Cu1—N1—C1—N2^i^	−0.7 (3)	C3—C4—C5—N1	0.8 (5)

Symmetry code: (i) −*x*, *y*, −*z*+1/2.

### Hydrogen-bond geometry (Å, º)

**Table:**

*D*—H···*A*	*D*—H	H···*A*	*D*···*A*	*D*—H···*A*
N2—H2*A*···Cl1^ii^	0.86	2.70	3.507 (2)	156
C2—H2···Cl1^iii^	0.93	2.77	3.482 (3)	134
C5—H5···S2^iv^	0.93	2.82	3.425 (3)	124

Symmetry codes: (ii) *x*, −*y*+1, *z*+1/2; (iii) *x*−1/2, −*y*+3/2, *z*−1/2; (iv) −*x*+1/2, *y*+1/2, −*z*+1/2.
